# Hippocampus of the APP^NL–G–F^ mouse model of Alzheimer’s disease exhibits region-specific tissue softening concomitant with elevated astrogliosis

**DOI:** 10.3389/fnagi.2023.1212212

**Published:** 2023-07-20

**Authors:** Chloe M. Hall, Soufian Lasli, Bianca Serwinski, Boris Djordjevic, Graham K. Sheridan, Emad Moeendarbary

**Affiliations:** ^1^Department of Mechanical Engineering, University College London, London, United Kingdom; ^2^School of Applied Sciences, University of Brighton, Brighton, United Kingdom; ^3^199 Biotechnologies Ltd., London, United Kingdom; ^4^Faculty of Social Sciences, Northeastern University London, London, United Kingdom; ^5^School of Life Sciences, University of Nottingham, Nottingham, United Kingdom

**Keywords:** Alzheimer’s disease, atomic force microscopy, brain tissue elasticity, healthy aging, hippocampus

## Abstract

Widespread neurodegeneration, enlargement of cerebral ventricles, and atrophy of cortical and hippocampal brain structures are classic hallmarks of Alzheimer’s disease (AD). Prominent macroscopic disturbances to the cytoarchitecture of the AD brain occur alongside changes in the mechanical properties of brain tissue, as reported in recent magnetic resonance elastography (MRE) measurements of human brain mechanics. Whilst MRE has many advantages, a significant shortcoming is its spatial resolution. Higher resolution “cellular scale” assessment of the mechanical alterations to brain regions involved in memory formation, such as the hippocampus, could provide fresh new insight into the etiology of AD. Characterization of brain tissue mechanics at the cellular length scale is the first stepping-stone to understanding how mechanosensitive neurons and glia are impacted by neurodegenerative disease-associated changes in their microenvironment. To provide insight into the microscale mechanics of aging brain tissue, we measured spatiotemporal changes in the mechanical properties of the hippocampus using high resolution atomic force microscopy (AFM) indentation tests on acute brain slices from young and aged wild-type mice and the APP^NL–G–F^ mouse model. Several hippocampal regions in APP^NL–G–F^ mice are significantly softer than age-matched wild-types, notably the dentate granule cell layer and the CA1 pyramidal cell layer. Interestingly, regional softening coincides with an increase in astrocyte reactivity, suggesting that amyloid pathology-mediated alterations to the mechanical properties of brain tissue may impact the function of mechanosensitive astrocytes. Our data also raise questions as to whether aberrant mechanotransduction signaling could impact the susceptibility of neurons to cellular stressors in their microenvironment.

## Introduction

The local mechanical properties of central nervous system (CNS) tissue are known to regulate cell behavior, particularly during embryonic brain development (Heisenberg and Bellaïche, 2013; Koser et al., 2016). For example, tissue stiffening coordinates brain morphogenesis at early stages of development (Barriga et al., 2018). In addition, mechanosensitive retinal ganglion cell (RGC) growth cones are guided by both biochemical (chemotaxis) and physical cues (durotaxis) as the RGC axons migrate through distinct layers of brain tissue (Koser et al., 2016). Studies have also shown that the mouse hippocampus becomes stiffer from infancy to adulthood (Elkin et al., 2009; Antonovaite et al., 2021a; Ryu et al., 2021). Beyond developmental stages, the mechanical properties of human brain tissue undergo significant changes as a result of both physiological and pathological aging (Sack et al., 2009; Levy Nogueira et al., 2016; Hiscox et al., 2018, 2020; Delgorio et al., 2021). Recent magnetic resonance elastography (MRE) studies indicate that, on a global macroscopic scale, cerebral tissue softens with age (Hiscox et al., 2018, 2021). However, the spatial resolution of MRE is limited (>1 mm) and it is thus less sensitive than atomic force microscopy (AFM) at detecting the mechanical characteristics of brain tissue at a cellular level (∼10 μm). It has also been challenging to obtain MRE measurements from deep subcortical limbic brain structures, such as the hippocampus, a region of the brain important for learning and memory, although significant advances have been made in recent years (McIlvain et al., 2018; Daugherty et al., 2020; Delgorio et al., 2021). Consequently, it is important to measure, at a cellular level, how the mechanical properties of distinct subregions of hippocampal brain tissue change from young adulthood to old age, particularly in the dentate gyrus, for example, where life-long neurogenesis takes place (Kuhn et al., 1996, 2018; Ben Abdallah et al., 2010). The structure of the hippocampus is unique and consists of a tri-synaptic neural network that processes novel and familiar sensory stimuli and generates new memory traces. The dentate gyrus (DG) is important for “pattern separation” of neuronal inputs from the cortex, i.e., a process that minimizes overlap in the neuronal ensembles activated by similar sensory experiences (Nakashiba et al., 2012). The CA3 region, on the other hand, is important for pattern completion, i.e., a process that facilitates the retrieval of a memory trace from partial activation of a particular neuronal ensemble (Rolls, 2013). The CA1 region is also critical for both the encoding and retrieval of hippocampal-dependent memories, e.g., population coding of place cell activity and generating spatial maps of new environments (Rolls, 2013). Interestingly, neurodegeneration and cell loss are not uniform across the distinct hippocampal cell layers in Alzheimer’s disease, with the CA1 region often reported as the most vulnerable to amyloid plaque toxicity (West et al., 2000; Kerchner et al., 2012). To investigate the mechanical effects of insoluble amyloid plaques in the hippocampus, we used the APP^NL–G–F^ mouse model because it closely mimics the amyloid pathology often present in humans with Alzheimer’s disease. Here, we employed high resolution AFM indentation tests on fresh mouse brain slices to explore how aging and amyloid pathology impact the mechanical properties of the hippocampus and whether amyloid beta (Aβ_42_)-induced astrogliosis coincides with tissue softening, an observation we have previously noted in traumatic injury-mediated glial scarring of the rat cortex (Moeendarbary et al., 2017).

## Results

**FIGURE 1:**
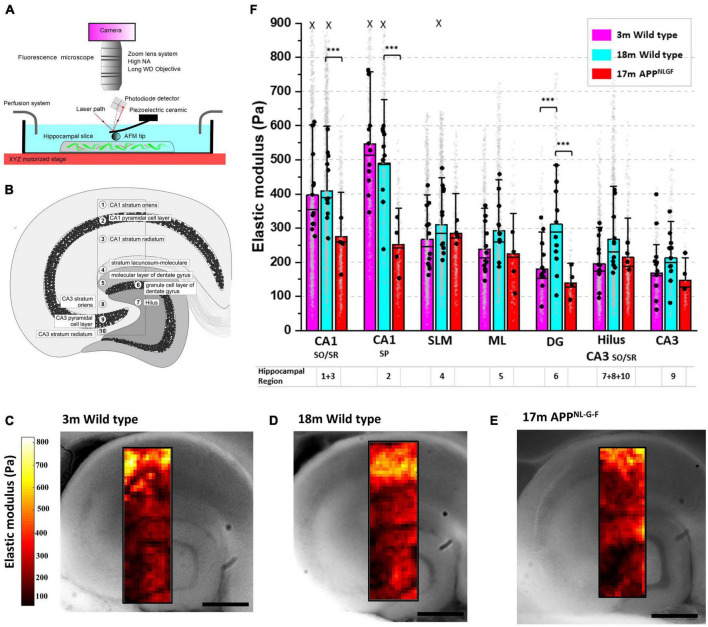
Hippocampal CA1 and dentate gyrus soften in aged mice with Alzheimer’s disease like amyloid pathology. **(A)** Schematic of the combined AFM, upright camera, and perfusion chamber set-up used to measure the microscale stiffness of acute hippocampal slices. **(B)** Cross section of the medial hippocampus showing the 10 subregions and the locations from which stiffness maps were extracted. **(C–E)** Representative hippocampal stiffness maps of 3-month wild-type in **(C)**, 18-month wild-type in panel **(D)** and 17-month APP^NL–G–F^ in panel **(E)**. The elastic moduli of individual indentation points are color-coded, with dark red colors representing softer areas and bright yellow colors depicting stiffer tissue regions. Scale bars = 450 μm. **(F)** Stiffness of the hippocampal substructures depicted in panel **(B)**. The stratum oriens/radiatum (SO/SR) of the CA1 are grouped and represented as regions 1 and 3, respectively. The Hilus, CA3 stratum oriens and CA3 stratum radiatum are also grouped and represented by the numbers 7, 8, and 10, respectively. The stratum lacunosum-moleculare (SLM) is represented by 4, and the molecular layer (ML) of the dentate gyrus is represented by 5. Finally, the three main neuronal cell body regions of the hippocampus, i.e., the granule cells of the dentate gyrus (DG), and the pyramidal cells of the CA3 and CA1 regions, are represented by the numbers 6, 9, and 2, respectively. AFM experiments were conducted on hippocampal slices from 3-month wild-type (*n* = 12), 18-month wild-type (*n* = 11), and 17-month APP^NL–G–F^ mice (*n* = 4). The vertical bars represent the means, the horizontal lines represent the medians, and the whiskers are the standard deviations of all experimental data points. Gray dots represent the individual data points for each AFM measurement. The black dots represent the average of the AFM measurements for each animal. X indicates the absence of some data points larger than 800 Pa (outliers were less than 0.5% of all data points). ***p < 0.001.

To investigate how age-related neurodegenerative pathologies cause region-specific changes in brain tissue stiffness, we quantified the elastic modulus of hippocampal tissue in response to both “healthy aging” (3- and 18-month wild types) and to Alzheimer’s disease-like pathology (at 17 months), using the APP^NL–G–F^ mouse model. APP^NL–G–F^ mice express amyloid precursor protein (APP) at similar levels and cell-type specificity to wild-type mice, but the combined effects of the triple mutations cause elevated levels of pathogenic amyloid beta (Aβ_42_) production and deposition (Saito et al., 2014). Using an upright light microscope and an AFM positioned on a motorized XY-stage (Figure 1A), we generated high resolution (30 μm steps) mechanical maps of freshly cut medial hippocampal slices of 500 μm thickness. To maintain the viability of *ex vivo* tissue, acute brain slices were continuously perfused with oxygenated artificial cerebrospinal fluid (aCSF) throughout the AFM indentation tests. Mechanical maps (1,500 × 450 μm) that spanned ten clearly identifiable subregions of the hippocampus (Figure 1B) were generated for comparisons between age and genotype. Cell body dense areas of the hippocampus (i.e., the pyramidal and granule cell bodies) were labeled as regions 2, 6, and 9 (Figure 1B) and were analyzed separately to the molecular layer of the dentate gyrus (region 5) and the stratum lacunosum-moleculare (region 4). The stratum oriens (SO, region 8) and stratum radiatum (SR, region 10) of the CA3 were grouped with the Hilus (region 7) to avoid any ambiguity in the demarcation of cell layers. Similarly, the SO (region 1) and SR (region 3) of the CA1 were grouped for analysis because, although the stratum oriens layer is ∼30% stiffer than the SR layer, their mechanical responses to both aging and AD-like pathology were very similar in magnitude.

Microscale stiffness maps of the hippocampus (Figures 1C–E) revealed its mechanical heterogeneity and the pattern of tissue elasticity closely corresponded to distinct functional and anatomical subregions. Focusing on the neuronal cell body regions (2, 6, and 9) of 3-month wild-types, we found that the CA1 pyramidal layer (546 Pa) was significantly stiffer than both CA3 pyramidal cells (168 Pa, *p* < 0.0001, Tukey contrast on the estimated marginal means from a linear mixed model) and dentate granule cell bodies (180 Pa, *p* < 0.0001) (Figure 1F). Similarly, the stratum oriens/radiatum regions of the CA1 (averaged at 396 Pa) were significantly stiffer than the Hilus and CA3 SO/SR regions combined (196 Pa, *p* < 0.0001). We next investigated if the mechanical properties of each hippocampal subregion changed with age (Figures 1C, D). For most of the hippocampal areas, AFM stiffness values did not differ significantly between aged 18-month and young 3-month-old wild-type mice. However, the stiffness of the dentate granule cell body layer increased by 73% in 18-month compared to 3-month old mice (311 Pa versus 180 Pa, respectively, *p* = 0.0025). This increase in stiffness in the dentate granule cell body layer with “healthy aging” was not observed in 17-month APP^NL–G–F^ mice (139 Pa). Indeed, stiffness measurements from aged APP^NL–G–F^ mouse brains were more similar to 3 month wild-types than to 18-month wild-type controls (Figures 1D, E), suggesting that the mechanisms that regulate DG granule cell layer stiffening in healthy aging are dysregulated in APP^NL–G–F^ mice. A potential limitation of our study was the omission of a 3-month old APP^NL–G–F^ group of mice to directly compare with hippocampal slices from the 3-month wild type mice. It is worth noting that the genetic background of the APP^NL–G–F^ mice is C57BL/6, i.e., the same as the wild type mice used here. At this young age, APP^NL–G–F^ mice do not display significant amounts of amyloid plaques in the cortex or hippocampus (Saito et al., 2014) and, therefore, we do not expect to see any appreciable differences in hippocampal mechanical properties between 3-month APP^NL–G–F^ mice and C57BL/6 wild type controls, although we cannot rule out that possibility. In addition to significant softening of the stratum oriens/radiatum regions of the CA1 (409 Pa versus 275 Pa in aged wild-type and APP^NL–G–F^ mice, respectively, *p* = 0.0026), the stiffness of the CA1 pyramidal cell layer also dropped by 49% (491 Pa in 18-month wild-types versus 251 Pa in APP^NL–G–F^ mice, *p* < 0.0001) (Figure 1F).

**FIGURE 2:**
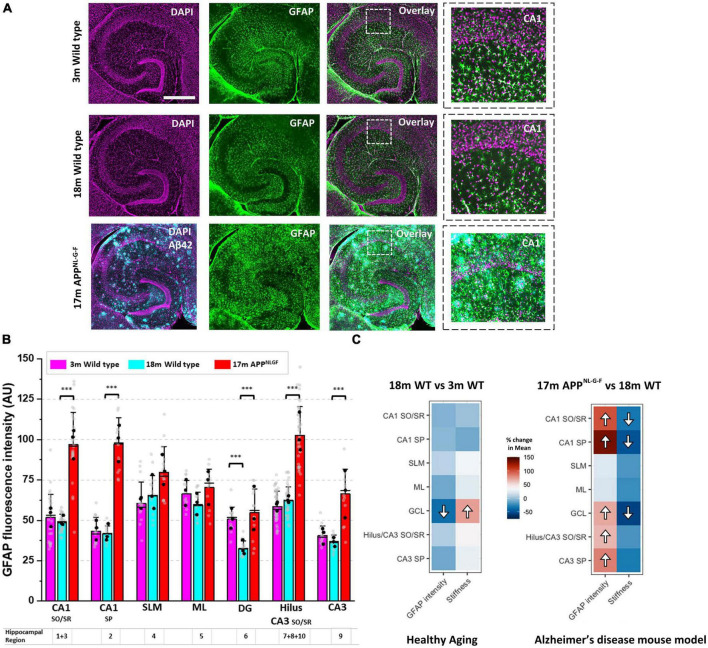
A drop in hippocampal tissue stiffness coincides with AD-induced astrogliosis. **(A)** Hippocampal slices from 3-month to 18-month wild-type mice were immunofluorescently labeled for glial fibrillary acidic protein (GFAP, green) and nuclei counterstained with DAPI (magenta). Slices from 17-month APP^NL–G–F^ mice were triple-stained for Aβ_42_ (cyan) highlighting the presence of amyloid plaques scattered throughout the hippocampus. Scale bar = 450 μm. Zoomed images of the CA1 regions are displayed on the far right to highlight changes in GFAP expression with age and with AD-like pathology. **(B)** GFAP fluorescence intensity was quantified in the ten hippocampal subregions and grouped as described in Figure 1B. The quantification of GFAP fluorescence intensity has been conducted in slices from *n* = 3 mice for 3-month wild-type, 18-month wild-type, and 17-month APP^NL–G–F^. The vertical bars represent the means, the horizontal lines represent the medians, and the whiskers are the standard deviations of all experimental data points. Gray dots represent all individual ROI fluorescence data points measured in each region. The black dots represent the average of regional ROI measurements for each animal. **(C)** The percentage (%) change in the mean elastic modulus and % change in GFAP fluorescence intensity in each hippocampal region (i.e., in 18-month wild-type mice versus 3-month wild-type, and 17-month APP^NL–G–F^ versus 18-month wild-type mice) are displayed as heatmaps. The significant differences found in Figure 1F and panel **(B)** have been highlighted with directional arrows for clarity. As the heatmaps indicate, in general, as hippocampal tissue stiffness decreases, GFAP fluorescence intensity increases and vice versa. ***p < 0.001.

We have previously shown that following a CNS injury, significant changes to brain and spinal cord tissue mechanics correlates strongly with astrogliosis (Moeendarbary et al., 2017). Therefore, we investigated whether the same relationship exists during pathophysiological brain aging. Employing a nuclear counterstain (DAPI) and antibodies targeting glial fibrillary acidic protein (GFAP), we measured changes in astrocyte reactivity in each subregion of the hippocampus. GFAP fluorescence intensity remained stable in most regions of the hippocampus from 3-months to 18-months of age in wild-type mice (Figure 2A), apart from the dentate granule cell layer (region 6) where it decreased (Figure 2B). Interestingly, this decrease in GFAP fluorescence intensity coincided with an increase in tissue stiffness (Figure 1F). Strikingly, when comparing GFAP expression in aged APP^NL–G–F^ mice versus aged wild-type mice (Figure 2A), we found a significant increase in GFAP expression in most hippocampal areas of APP^NL–G–F^ mice (Figure 2B), particularly in the Hilus/CA3 (regions 7, 8, 9, 10) and CA1 (regions 1, 2, 3) (Figure 2B). To highlight this relationship between elevated astrogliosis and brain tissue softening, we generated two heatmaps (Figure 2C) that clearly illustrate this phenomenon. In summary, the heatmaps show that with healthy aging, there is a decrease in GFAP-labeled astrocytes in the dentate granule cell layer coinciding with an increase in tissue stiffness. Moreover, in APP^NL–G–F^ mice that display widespread amyloid plaque pathology in the hippocampus, there are significant increases in GFAP expression in the CA1, CA3, and DG regions and this coincides with a reduction in the elastic modulus of the CA1 and DG hippocampal areas. The slight softening of the CA3/Hilus regions in APP^NL–G–F^ mice compared to 18-month wild-types were not significant, potentially due to the CA3 and Hilus being relatively soft areas of the hippocampus.

## Discussion

Injuries to the brain and spinal cord can lead to significant and sustained softening of CNS tissue lasting several weeks (Moeendarbary et al., 2017). Moreover, softening of the damaged tissue regions directly correlates with the formation of a glial scar, measured as a significant upregulation in glial fibrillary acidic protein (GFAP) levels, which is an indicator of astrogliosis (Yang and Wang, 2015). While the effects of glial scars on functional recovery is a matter of controversy (Anderson et al., 2016; Yang et al., 2020), CNS injury-associated tissue softening most likely impacts the extent of neural regeneration, since neural stem cell fate and the intrinsic properties of neurons can be influenced by the mechanical properties of the extracellular microenvironment (Georges et al., 2006; Pathak et al., 2014; Zhang et al., 2014; Ucar et al., 2021). The mechanical maps of the hippocampus presented here highlight substantial softening (and possibly impaired tissue stiffening mechanisms) of specific regions of the hippocampus that contain the cell bodies of neurons known to be important for learning and memory formation. It is perhaps unsurprising that amyloid pathology differentially impacts the mechanical properties of distinct subregions of the hippocampus given their functional specialization and contrasting responses to various toxic insults (West et al., 2000; Mao et al., 2013; Rolls, 2013; Daugherty et al., 2020; Gonzalez-Rodriguez et al., 2021). The CA1 pyramidal neurons, for example, are more vulnerable than dentate granule cells to glutamate-mediated excitotoxicity (Butler et al., 2010). As such, several studies have reported that the CA1 region displays more cell loss and a greater reduction in volume than the dentate gyrus in patients with Alzheimer’s disease (Bobinski et al., 1997, 1998). Here, our measurements show that the CA1 region softens in aged APP^NL–G–F^ mice when compared to both young and aged wild-type mice. This is consistent with indentation studies in the cortex of APP/PS1 mutant mice (Menal et al., 2018), MRE studies in mice (Munder et al., 2018), and with MRE experiments in humans with Alzheimer’s disease (Murphy et al., 2011; Arani et al., 2015; Hiscox et al., 2020) that report softening of cortical brain regions. Interestingly, the softest hippocampal region (139 Pa) measured from all groups and ages was the DG cell layer of 17-month APP^NL–G–F^ mice. The mechanical properties of APP^NL–G–F^ mouse dentate granule cells were more similar to young 3-month wild-type mice (180 Pa) than to 18-month wild-types (311 Pa). These results are in contrast to a recent study that used the APP/PS1 mouse model and found that amyloid pathology increased the stiffness of the hippocampus (Antonovaite et al., 2021b). However, the APP/PS1 mice were 6-months of age and, therefore, not comparable to the old age mice used here. This does, however, raise the question of whether amyloid pathology causes transient stiffening of the hippocampus in middle-age (∼6–9 months in mice), followed by tissue softening in old age (>15 months in mice). This hypothesis is supported by magnetic resonance elastography results in aged APP/PS1 mice where the brain was measured to be softer than wild-type mice (Murphy et al., 2012). For a more extensive review of the literature surrounding changes in brain tissue stiffness during brain maturation, “healthy aging,” and Alzheimer’s disease, we refer the reader to more comprehensive review articles (Levy Nogueira et al., 2016; Hall et al., 2021; Hiscox et al., 2021; Coelho and Sousa, 2022; Donnaloja et al., 2023; Feng et al., 2023).

In the present study, we found that 17 month-old APP^NL–G–F^ mice, which have substantial amyloid pathology (Figure 2A), display significant astrogliosis in the Hilus, CA3 and CA1 regions of the hippocampus, consistent with observed gliosis in human AD patients (Gonzalez-Rodriguez et al., 2021). Interestingly, the astrogliosis coincides with tissue softening although a direct cause-and-effect cannot be inferred from these observations. It is possible that the mechanical properties of astrocytes are different in mice with AD-like amyloid pathology compared to healthy wild-type mice. However, we also observed a similar phenomenon with healthy aging in the dentate granule cell body layer, i.e., tissue stiffening from 3- to 18-months in wild-type mice coincided with a decrease in astroglial reactivity (Figure 2C). The dentate granule cell body layer is home to the subgranular neurogenic niche where active neurogenesis continues into late adulthood and old age (Kuhn et al., 2018), although rates of neurogenesis are known to decline drastically with age (Kuhn et al., 1996; Ben Abdallah et al., 2010). Therefore, as we age, neural stem cells are continually generated and differentiate into mature neurons that migrate to the outer layers of the dentate gyrus, and thus cause this tightly packed layer of cell bodies to grow and widen over time. This is in contrast to the CA3 and CA1 pyramidal cell layers, where little or no neurogenesis takes place in adulthood (Schinder and Gage, 2004).

One factor that may contribute to hippocampal tissue softening in the brain of APP^NL–G–F^ mice is neuroinflammation-mediated hypertrophy of astrocytes which is clearly evident around amyloid plaques (Figure 2A). It has been demonstrated, using scanning force microscopy, that astrocytes are softer than neuronal cells (Lu et al., 2006) and so if there is a shift in the ratio of astrocytes to neurons in areas of the hippocampus susceptible to amyloid plaque-mediated excitotoxicity, this could help to explain a drop in the elastic modulus of the CA1 region in APP^NL–G–F^ mice. Others have shown in cell culture systems that traumatic mechanical injury of astrocytes leads to upregulation of GFAP and a softening of non-nuclear regions of the astrocyte syncytium (Miller et al., 2009). Interestingly, the authors could inhibit the softening of injured astrocytes by non-selectively blocking P2-type purinergic receptors using the antagonist PPADS. This suggests that stretch-induced mechanical injury can lead to ATP release from astrocytes and that the reactive astrogliosis and subsequent softening of glial cells is dependent on purinergic receptor signaling. Aβ_42_ has also been shown to cause the release of ATP from astrocytes and this could be a neuroprotective mechanism that leads to attenuation of synapse loss and inhibits Aβ_42_-mediated reduction of long-term potentiation (LTP) in hippocampal slices (Jung et al., 2012). Therefore, astrocytic hypertrophy and glial cell softening may be a neuroprotective response to amyloid plaque deposition in the Alzheimer’s disease brain (Perez-Nievas and Serrano-Pozo, 2018). Because neurons grown in culture appear to favor very soft substrates compared to other cell types and tend to display greater neurite extension and branching on soft compliant surfaces (Balgude et al., 2001; Flanagan et al., 2002; Discher et al., 2005; Lu et al., 2006), reactive astrocytes may provide a mechanical environment conducive to axonal regrowth or neural regeneration (Anderson et al., 2016). However, a mechanically-favorable tissue environment is not the only element needed to encourage neuronal repair. Brain parenchyma laden with amyloid plaques is also full of soluble cell signaling factors that are inhibitory to neural regeneration and axonal repair (Hoozemans et al., 2006; Asik et al., 2021). The Alzheimer’s disease brain is known to express high levels of neuroinflammatory markers and mouse models of AD recapitulate this phenotype well by displaying enhanced microglial reactivity in cortical and hippocampal regions (Varnum and Ikezu, 2012; Edler et al., 2021). Therefore, neuroinflammation present in the APP^NL–G–F^ mouse hippocampus may lead to ATP-dependent reactive astrogliosis, particularly close to amyloid plaque deposits. Delekate et al. (2014) have shown that astrocytes near to amyloid plaques are hypertrophic in the APP/PS1 mouse model of AD and that astroglial reactivity is inhibited by P2-type purinergic receptor blockade but enhanced by an increase in cortical adenosine diphosphate (ADP) concentration. They went on to show that blocking the P2X receptor has little effect, but inhibition of P2Y1 receptors which are highly expressed by hypertrophic astrocytes that border amyloid plaques, decreases astrocyte reactivity. Whilst we have not shown a link here between aberrant ATP release from astrocytes and decreases in brain tissue stiffness in the APP^NL–G–F^ mouse, we highlight this a potential molecular mechanism that warrants further investigation by research groups aiming to identify potential new drug targets for Alzheimer’s disease.

Moreover, further research is required to investigate the full spectrum of temporal changes in hippocampal mechanics throughout the life course of Alzheimer’s disease mouse models. Alternatively, it is also possible that there are significant differences in brain tissue mechanics between transgenic AD-mouse models (e.g., APP/PS1) and knock-in AD-mouse models (e.g., APP^NL–G–F^). The latter knock-in models should, in theory, recapitulate the human version of the disease more faithfully. Moreover, it will be important to interrogate the functional significance of aberrant mechanical properties of the DG and CA1 neuronal cell layers, which we predict will likely impact mechanotransduction signaling in hippocampal neurons and glial cells given the expression of mechanoreceptors in these cells (Zhang et al., 2014; Velasco-Estevez et al., 2018; Chi et al., 2022). Since the extracellular matrix (ECM) is known to be important in regulating synaptic plasticity and may be important in maintaining the structural integrity (or stiffness) of aged brain tissue, it is possible that softening of the CA1 and DG neuronal cell layers in Alzheimer’s disease disrupts the ability of the hippocampus to process sensory stimuli and encode new memories (Dityatev et al., 2010).

We are only beginning to understand how physiological and disease-related perturbations to the mechanical properties of CNS tissue impact brain development, cellular responses to injury, brain aging, and cognitive decline (Hall et al., 2021; Donnaloja et al., 2023). For example, it has recently been shown that mechanical stimulation of presynaptic boutons increases glutamate release in the rat hippocampus (Ucar et al., 2021). This suggests that mechanical changes may contribute to altered neuronal signaling and, therefore, may conceivably contribute to the characteristic cognitive deficits present in people affected by Alzheimer’s disease (Huff et al., 1987). Interestingly, deletion of the mechanically-gated ion channel, Piezo1, from astrocytes causes a reduction in hippocampal volume and impairs long-term potentiation (LTP) of synaptic transmission (Chi et al., 2022). The results presented here are also important in the context of *in vitro* research that uses dissociated cultures of neurons and glial cells because, as we and others have shown (Georges et al., 2006; Moshayedi et al., 2010; Damani et al., 2011; Zhang et al., 2014; Wen et al., 2018; Kayal et al., 2020), the mechanical microenvironment of cells impacts their morphology and functions. Therefore, it is important to recapitulate the mechanical environment of the brain when creating novel *in vitro* models of neuron/glial crosstalk.

Finally, it should be noted that atomic force microscopy is a contact-based method to measure cellular forces and the mechanical properties of tissue, and it is unlikely that AFM measurements on living humans will be possible any time soon. However, the findings presented here raise novel clinically-relevant questions such as why amyloid pathology-driven changes to brain tissue mechanics coincides with increased levels of astrogliosis in the aging brain. Moreover, recent advances in the spatial resolution of MRE (Hiscox et al., 2018; McIlvain et al., 2018; Daugherty et al., 2020; Delgorio et al., 2021; Morr et al., 2022) suggest that it may be possible in the near future to study the mechanical signature of specific brain structures, such as the CA1 region of the hippocampus, and use it as a diagnostic tool to detect early (or advanced pre-clinical) signs of neurodegenerative disease, thus facilitating more accurate diagnosis and treatment of patients with dementia.

## Materials and methods

### Animals

All animal work was carried out in accordance with the UK Animals (Scientific Procedures) Act 1986. Female C57Bl/6 (3- and 18-month wild-types) or female APP^NL–G–F^ (17-months) mice were group housed until age of use. APP^NL–G–F^ mice were investigated at 17 months of age, a time-point at which they displayed advanced Alzheimer’s disease-like amyloid pathology in the hippocampus and an age which could be directly compared to the 18-month healthy aged wild-type group. The number of mice used for AFM experiments were as follows, 3 month wild-type (*n* = 12), 18-month wild-type (*n* = 11), and 17-month APP^NL–G–F^ mice (*n* = 4). For immunofluorescence experiments, animal numbers were *n* = 3 for each mouse group. Mice were sacrificed by cervical dislocation, followed by permanent cessation of the circulation by decapitation. The brain was subsequently dissected out and placed into ice-cold artificial cerebrospinal fluid (aCSF). The composition of aCSF was 125 mM sodium chloride, 2.4 mM potassium chloride, 26 mM sodium bicarbonate, 1.4 mM sodium phosphate, 20 mM d-glucose, 3 mM magnesium chloride, and 0.5 mM calcium chloride. The cerebellum and olfactory bulbs were removed using a scalpel, and the brain bisected along the midline. A cut 10 degrees into the left hemisphere was performed to remove the dorsal cortex, and transverse sections were taken along the long axis of the hippocampus. This exposed the surface of the brain which was then glued, using cyanoacrylate (“superglue”), to the metal vibratome stage, and covered with ice-cold aCSF. Hippocampal brain sections of 500 μm thickness were cut using a Campden Instruments 7000smz vibratome (Campden Instruments, London, UK), and left to recover in aCSF perfused with 95% O_2_ and 5% CO_2_ at 22°C. The right hemisphere of the brain was fixed in cold 10% formalin solution for subsequent immunofluorescence experiments.

### Atomic force microscopy

One 500 μm brain slice was placed into a 35 mm petri dish and held in position using a glass coverslip with a 4 mm diameter hole in the center which was positioned over the hippocampus. AFM indentation measurements were performed with a JPK Nanowizard Cellhesion 200 (JPK Instruments AG, Berlin, Germany) placed on an optical microscope with a motorized xy stage. Cantilevers with pyramidal tips (MLCT, Bruker) were used, with a nominal spring constant of 0.07 N/m. The actual spring constant was measured using the thermal noise method. A 25 μm glass bead (Cospheric, Goleta, CA, USA) was attached to the cantilever tip using ultraviolet curing glue. Force-distance curves were taken with an approach speed of 10 μm/s and a set force of 15 nN every 30 μm, across an area of 1,500 × 450 μm, spanning the CA3, dentate gyrus and CA1 regions of the hippocampus. During experiments, the brain slice was continually perfused with oxygenated aCSF at a flow rate of 30 mL/h. All experiments were concluded within 8 h post-decapitation.

### AFM data analysis

Elastic moduli values (E) of hippocampal brain tissue were extracted from AFM force-indentation curves by fitting the contact portion of the curve to a Hertz contact model between a sphere and an infinite half space in JPK data processing software. Below is the Hertz equation, where *R*_*c*_is the radius of the sphere used for indention, i.e., 12.5 μm.


$$F=\frac{\sqrt[{4}]{R_{c}}}{3}\,\frac{E}{1-v^{2}}\,\delta^{3/2}$$

Poisson’s ratio (ν) was set to 0.5 (Esteki et al., 2020; Javanmardi et al., 2021). The indentation depth (δ) in our experiments was typically 5–15 μm but force-distance curves were only analyzed up to 7 μm indentation depths to fit the requirements of the Hertz model, where the radius of contact area (α) or indentation depth need to be smaller than the size of the indenter. We expect some small deviations in the estimation of the exact elastic modulus due to using a simple Hertz model in our analysis, different sources of nonlinearity such as the experimental condition of large deformations, and the inherent nonlinearities associated with soft tissues. However, the slight uncertainty in the absolute value of elastic modulus does not impact the trends we observe and the generality of our findings, especially considering the magnitude of differences, which we have discussed as percentages. Elasticity maps of the Young’s modulus values at x and y coordinates were created in MATLAB. Elasticity maps were then overlaid with images taken during the experiment using an upright microscope (GXMZ monozoom) and CCD camera (Hamamatsu Orca-ER). The cantilever was visible in these images, and so exact positions of indentation could be noted allowing for accurate sampling of elastic modulus of hippocampal subregions. Young’s modulus values per subregion were grouped using a custom script in MATLAB (Mathworks). Ten distinct hippocampal cell layers were chosen for analysis (Figure 1B). Cell layers 1–3 are part of the CA1 region and contain the dendrites (stratum oriens), the cell bodies (stratum pyramidale) and the axons (stratum radiatum) of the CA1 principal neurons. Cell layer 4, the stratum lacunosum moleculare contains GABAergic interneurons and myelinated axon projections from the entorhinal cortex. Cell layer 5, the molecular layer of the dentate gyrus (DG) contains the dendritic fields of the DG granule cells and cell layer 6, the dentate granule cell layer, contains the cell bodies. Cell layer 7 is the Hilus, a region densely packed with GABAergic interneurons. Cell layers 8–10 compose the CA3 region. Cell layer 8, the CA3 stratum oriens, contains mossy fiber axons from the DG granule cells. Cell layer 9 contains the cell bodies of CA3 pyramidal neurons. Finally, cell layer 10, the CA3 stratum radiatum, largely contains populations of interneurons. Subregions of the CA3 and CA1 were grouped according to whether they predominantly contained principal neuronal cell bodies or their axons/dendrites. The hilus was grouped with the CA3 SO/SR regions due to continuity between the CA3 SR and hilus, and similarity in tissue composition.

### Immunofluorescence

Brain hemispheres that had been formalin-fixed and cryopreserved were sliced into 40 μm free-floating sections in phosphate-buffered saline (PBS) using the Campden Instruments 7000smz vibratome. Brain sections were washed, blocked and permeabilized in PBS with 0.3% Triton X and 10% normal goat serum (Abcam, ab7481) for 1 h at 22°C. Chicken anti-GFAP antibody (Abcam, ab4674) and rabbit anti-Amyloid 1-42 (Abcam, ab201061) were diluted (1:800) in PBS with 0.3% Triton X and 2% normal goat serum and added to the brain sections for 24 h at 4°C. Samples were then washed and incubated with secondary Dylight 550 goat anti-rabbit (Thermo Fisher, SA510033) and Dylight 650 goat anti-chicken (Thermo Fisher, SA510073) antibodies at a 1:500 dilution for 3 h at 22°C. Slices were finally washed in PBS (3 × 5 min with gentle shaking) and mounted on superfrost plus microscope slides using Fluoroshield mounting medium with DAPI (Abcam, ab104139). Slides were left to dry overnight and then imaged using an epifluorescent Leica DMi8 microscope with a 10X (0.3 NA) objective. Individual images were stitched together to reconstruct a single image of the whole hippocampal slice.

### Image analysis

For quantitative GFAP fluorescence intensity analysis, all images were captured using the same Leica DMi8 microscope settings and images were exported as 8-bit tif files. Image analysis was conducted using the software package FIJI (Image J). Briefly, GFAP expression was quantified by manually selecting 5 regions of interest (ROI) of approximately 22,500 μm^2^ (150 × 150 μm) for each hippocampal cell layer. Using the functions “Analyze and Measure” in FIJI (Image J), the average GFAP fluorescence intensity of each ROI was calculated. Three brain sections from 3 different animals per age group/genotype were analyzed (*n* = 3). Therefore, 15 fluorescence intensity (ROI) measurements were captured for every hippocampal cell layer for each group of mice. To generate heatmaps for Figure 2C, the percentage (%) change in GFAP expression in 18 month mice relative to the 3 month control group, and the (%) change in GFAP expression in 17 month APP^NL–G–F^ mice relative to the 18 month wild type group, are represented as heatmap images alongside the corresponding % change in brain tissue elasticity for each region of the hippocampus (Figure 2C).

### Statistical analysis

Analyses and plotting were performed in Microsoft Office Excel, MATLAB (Mathworks) or Origin (OriginLab). A linear mixed effects model was performed on the data in R studio (version 1.4.1106). Normality of residuals was checked using Q-Q plots. Fixed effects were incorporated as hippocampal subregion and animal age or genotype group. Individual animals were included in the model as a random effect. *Post hoc* Tukey comparisons were performed on estimated marginal means from the linear regression model to give the stated statistical values in this test. Data were cleansed by removing outliers which were defined as data points lying ± 2 × standard deviations above or below the mean value (per mouse group and subregion). This was due to a very small number of data points being approximately 250% greater than the mean value or, on the other end of the scale, below 50 Pa, and so outliers were considered as experimental errors that may have been caused by uneven indentation sites on the surface of the brain slices, thus leading to “noisy” indentation curves.

## Data availability statement

The original contributions presented in this study are included in this article/supplementary material, further inquiries can be directed to the corresponding authors.

## Ethics statement

All experiments using animals were performed in accordance with the UK Animals (Scientific Procedures) Act 1986 and were approved by the University College London Animal Welfare and Ethical Review Board.

## Author contributions

GS and EM conceived the project, designed the research, and wrote the manuscript with contributions from CH and SL. CH and SL performed the experiments. CH, BS, BD, GS, and EM analyzed the data. All authors discussed the results.
