# Corrigendum: Hippocampus of the APP^NL−G−F^ mouse model of Alzheimer's disease exhibits region-specific tissue softening concomitant with elevated astrogliosis

**DOI:** 10.3389/fnagi.2023.1273549

**Published:** 2023-08-28

**Authors:** Chloe M. Hall, Soufian Lasli, Bianca Serwinski, Boris Djordjevic, Graham K. Sheridan, Emad Moeendarbary

**Affiliations:** ^1^Department of Mechanical Engineering, University College London, London, United Kingdom; ^2^School of Applied Sciences, University of Brighton, Brighton, United Kingdom; ^3^199 Biotechnologies Ltd., London, United Kingdom; ^4^Faculty of Social Sciences, Northeastern University London, London, United Kingdom; ^5^School of Life Sciences, University of Nottingham, Nottingham, United Kingdom

**Keywords:** Alzheimer's disease, atomic force microscopy, brain tissue elasticity, healthy aging, hippocampus

In the published article, there was an error regarding the affiliation for [Bianca Serwinski]. As well as having affiliation(s) [1 and 3] they should also have [Faculty of Social Sciences, Northeastern University London, London, United Kingdom].

The authors apologize for this error and state that this does not change the scientific conclusions of the article in any way. The original article has been updated.

